# Phylogeny and taxonomic revision of *Kernia* and *Acaulium*

**DOI:** 10.1038/s41598-020-67347-1

**Published:** 2020-06-25

**Authors:** Lei Su, Hua Zhu, Yongchun Niu, Yaxi Guo, Xiaopeng Du, Jianguo Guo, Ling Zhang, Chuan Qin

**Affiliations:** 1 NHC Key Laboratory of Human Disease Comparative Medicine, Institute of Medical Laboratory Animal Science, Chinese Academy of Medical Sciences (CAMS), Beijing, China; 2 Beijing Engineering Research Center for Experimental Animal Models of Human Critical Diseases, Chinese Academy of Medical Sciences (CAMS), Beijing, China; 3 Key Laboratory of Microbial Resources, Ministry of Agriculture/Institute of Agricultural Resources and Regional Planning, Chinese Academy of Agricultural Sciences, Beijing, 100081 China

**Keywords:** Fungal systems biology, Microbiology, Fungi

## Abstract

The genera *Kernia* and *Acaulium* comprise species commonly isolated from dung, soil, decaying meat and skin of animal. The taxonomy of these fungi has been controversial and relies mainly on morphological criteria. With the aim to clarify the taxonomy and phylogeny of these fungi, we studied all the available ex-type strains of a large set of species by means of morphological and molecular phylogenetic analyses. Phylogenetic analysis of the partial internal transcribed spacer region (*ITS*) and the partial 28S rDNA (*LSU*) showed that the genera *Kernia* and *Acaulium* were found to be separated in two distinct lineages in Microascaceae. Based on morphological characters and multilocus phylogenetic analysis of the *ITS*, *LSU*, translation elongation factor 1α and β-tubulin genes, the species in *Kernia* and *Acaulium* were well separated and two new combinations are introduced, i.e. *Acaulium peruvianum* and *Acaulium retardatum*, a new species of *Kernia* is described, namely *Kernia anthracina*. Descriptions of the phenotypic features and molecular phylogeny for identification are discussed for accepted species in two genera in this study.

## Introduction

*Kernia* was erected by Nieuwland^1^ for a group of fungi with cleistothecial, with *Kernia nitida* (Saccardo) Nieuwland as type species firstly and subsequent species have all been characterized by fascicled hair-like ascocarp appendages and reddish-brown to brown ascospores. In 1971, the ascomycete genus *Kernia* is emended to revised concepts by Malloch^2^. Five species, *K. bifurcotricha* Saxena & Mukerji, *K. hippocrepida* Malloch & Cain, *K. nitida* (Sacc.) Nieuwland, *K. hyalina* Malloch & Cain and *K. pachypleura* Malloch & Cain, are included and two species, *K, brachytricha* (Ames) Benjamin and *K. geniculotricha* Seth, are placed in synonymy with *K. nitida*. Besides, *K. bartlettii* (Massee & Salmon) Benjamin, *K. furcotricha* Tandon & Bilgrami, and *K. spirotricha* Benjamin, are excluded from *Kernia*. And some new species and combinations were added to it later as *K. ovata* (Booth) Malloch & Cain, *K. retardata* Udagawa & T. Muroi, *K. setadispersa*, *K. cauquensis*, *K. irregularis*, *K. peruviana* Udagawa & Furuya, *K. columnaris* (H.J. Swart) Woudenb & Samson^3–10^. In recently, *K. hyalina* is excluded from *Kernia* by phylogenetic analysis based on a combined *LSU* and *ITS* sequence dataset and morphological characteristics^11^. Although 11 species are accepted in *Kernia*, many described species are of doubtful identity because their type materials are lost and their protologues are uninterpretable.

The genus *Acaulium* was established as the sexual morph and the type species is *Acaulium albonigrescens* Sopp.^12^, and this genus was considered as congeneric with *Microascus*^13–15^. *Acaulium* is characterised by annellidic conidiogenesis, guttulate conidia and mycelium forming abundant hyphal fascicles and has generally been considered a synonym of *Scopulariopsis* but recently was re-instated as an accepted genus of Microascaceae with three species as *A. acremonium* (Delacr.) Sandoval-Denis, Guarro & Gené, *A. albonigrescens* Sopp, Skr. Vidensk.-Selsk and *A. caviariforme* (Malloch & Hubart) Sandoval-Denis, Guarro & Gené^16^. *Acaulium album*, formerly known as *Doratomyces putredinis*, is transferred to *Acaulium* and redescribed by Woudenberg^10^ based on morphological, physiological and molecular phylogenetic analyses. In addition, *A. pannemaniae* Sandoval-Denis is introduced in this genus by morphological and phylogenetic analyses of *LSU*^17^. Five species are currently accepted at present^10^.

*Kernia* currently comprises species that are commonly isolated from the dung of animal^2, 8–10^, except two species *K. retardata* and *K. peruviana*^8, 9^, which isolated from soil. *Acaulium* species have been reported from a variety of environments such as skin of a horse, decaying meat, soil and so on^10, 16^. From beginning, the genera of *Kernia* and *Acaulium* have been controversial and rely mainly on morphological criteria. Recent molecular studies have demonstrated that the Microascaceae contains several closely related genera and difficult to separate morphologically^16^. Multilocus phylogenetic analysis have considerably improved our understanding of species concepts in many fungal groups^18–25^, but the studies for revising the genera of *Kernia* and *Acaulium* are relatively limited. Besides, during our investigation of intestinal fungi in animals in China, three particular *Kernia* isolates from the dung of marmot were isolated. The present work also aims to clarify the taxonomic position of these strains as putative new species using the genealogical concordance analysis^24^. We provide a multigene (*ITS*, *LSU*, *TEF*, *TUB*) phylogeny of *Kernia* and *Acaulium* and related fungi based on a large set of strains, which includes all available ex-type cultures and well-identified reference strains from international culture collections.

## Results

### Generic circumscription

**Figure 1:**
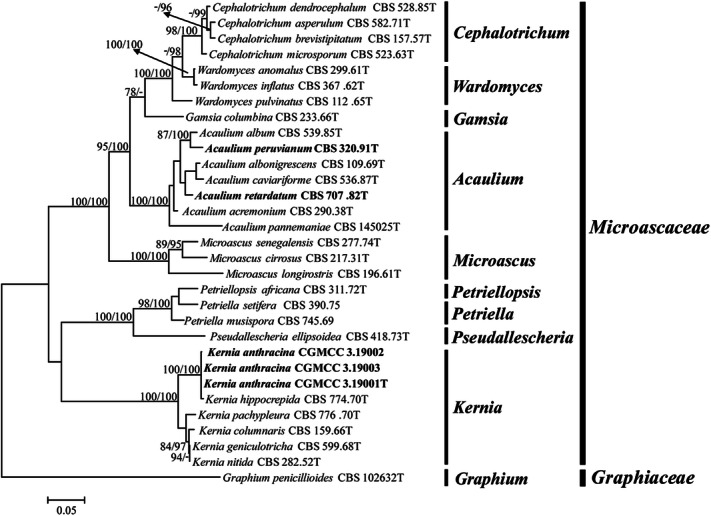
Maximum likelihood (ML) tree obtained from the combined *LSU* and *ITS* sequences of 31 representative taxa of Microascaceae and Graphiaceae. Numbers on the branches are ML bootstrap values (bs) above 75%, followed by Bayesian posterior probabilities (pp) above 95%. A dash (–) indicates support value lower than 75% bs or 95% pp. Branch lengths are proportional to distance. Ex-type strains are indicated with T. The tree was rooted to *Graphium penicillioides* (CBS 102632).

**Figure 2:**
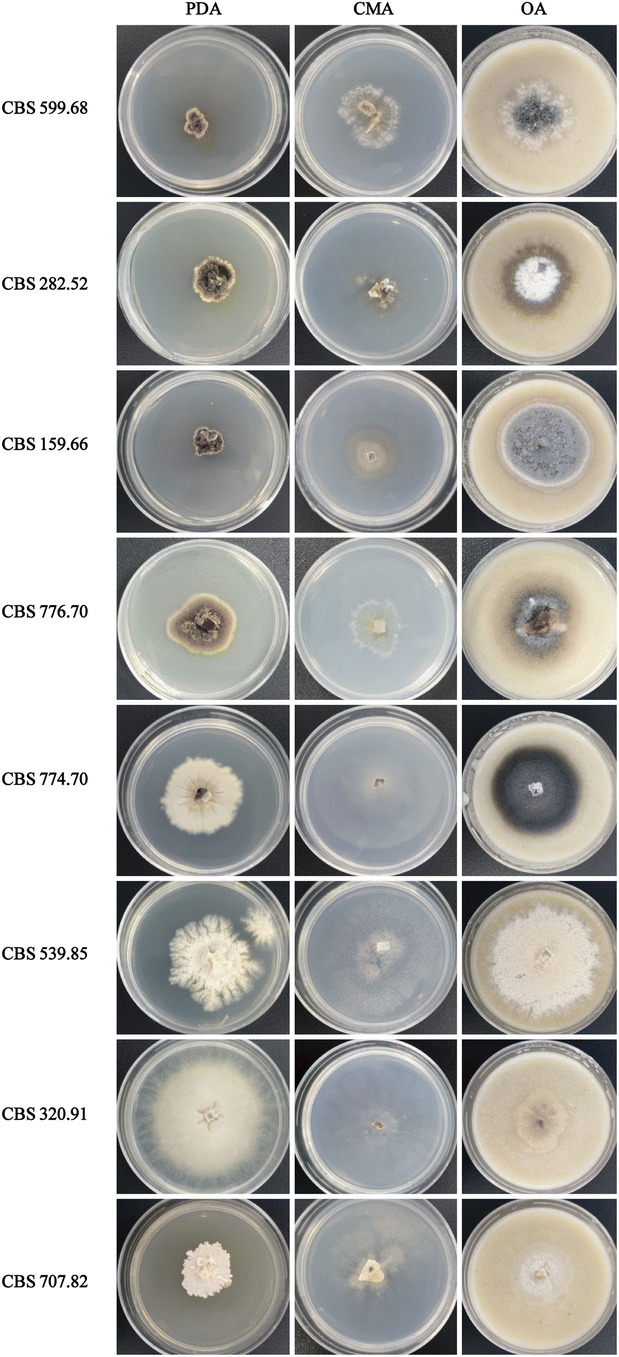
The colony morphology of the species in *Kernia* and *Acaulium* was growing on PDA, CMA and OA after 35 days, respectively.

DNA sequences determined in this study are deposited in GenBank, and accession numbers are listed in Table 1. To delineate generic boundaries, we conducted a phylogenetic analysis using the combined *LSU* and *ITS* datasets including 29 currently accepted species belonging to nine genera of Microascaceae and one species of the family Graphiaceae. *Graphium penicillioides* were selected as outgroup (Fig. 1). The final alignment consisted of 31 strains and contained 1,385 characters (*LSU* 796, *ITS* 589). Figure 1 shows the ML tree including ML bootstrap values (bs) and posterior probabilities (pp) values. The trees obtained from ML and Bayesian analyses of the individual loci and the combined analysis showed congruent topologies. The phylogenetic inferences (Fig. 1) showed that *Kernia* and *Acaulium* were monophyletic, the species of *Kernia* and *Acaulium* clustered into a single, well-supported lineage (bs = 100%/pp = 100%), respectively. Figure 2 is demonstrated that the colonies of *K. peruviana* CBS 320.91, *K. retardata* CBS 707.82 and *A. album* CBS 539.85 can form white to pale grey colonies with dense hyphal fascicles. Therefore, *K. peruviana* and *K. retardata* were identified as the *Acaulium* species in this study. However, other type *Kernia* species grow slowly and form compact, brown to dark brown colonies apparently different with the *Acaulium* species. Among with the *K. geniculotricha* and *K. nitida* were located in the same clade with the value of (bs = 94%/pp = 91%) and combined with morphological characters, they could be identified as the same species in this research. Three *Kernia* strains which were isolated from the dung of marmot, were clustered with the species of *K. hippocrepida* and have a well-supported value (bs = 100%/pp = 100%).

**Table 1:** Strains and sequence accession numbers included in this study.

Species	Strain no.	Isolation source	Location	GenBank accession no.			
				ITS	TUB	LSU	TEF
*Acaulium acremonium*	CBS 290.38T	Skin of a horse	Denmark	LM652456	LN851108	LN851001	HG380362
*Acaulium albonigrescens*	CBS 109.69T	Litter, treated	Japan	KY852469	LN851111	KY852480	LN851058
*Acaulium album*	CBS 539.85T	Hair in dung in pole cat	Netherlands	**MN991960**	**MN982419**	**MN991968**	**MN982411**
*Acaulium caviariforme*	CBS 536.87T	Decaying meat	Belgium	LM652392	LN851112	LN851005	LN851059
*Acaulium pannemaniae*	CBS 145025T	Soil	Netherlands	LS999990	LS999993	LS999991	LS999992
*Acaulium peruvianum*	CBS 320.91T	Soil	Peru	**MN991959**	**MN982418**	**MN991966**	**–**
*Acaulium retardatum*	CBS 707.82T	From paddy soil	Japan	**MN991961**	**–**	**MN991969**	**MN982412**
*Cephalotrichum asperulum*	CBS 582.71T	Soil	Argentina	LN850960	LN851114	LN851007	LN851061
*Cephalotrichum brevistipitatum*	CBS 157.57T	*Solanum tuberosum*	Netherlands	LN850984	LN851138	LN851031	LN851084
*Cephalotrichum dendrocephalum*	CBS 528.85T	Cultivated soil	Iraq	LN850966	LN851120	NG_059041	LN851067
*Cephalotrichum microsporum*	CBS 523.63T	Wheat field soil	Germany:	LN850967	LN851121	LN851014	LN851068
*Gamsia columbina*	CBS 233.66T	Sandy soil	Germany	LN850990	LN851147	LN851039	LN851092
*Graphium penicillioides*	CBS 102632T	*Populus nigra*	Czech Republic	KY852474	**–**	KY852485	**–**
*Kernia anthracina*	CGMCC 3.19001T	Dung of marmot	Beijing, China	**MK773539**	**MK773545**	**MK773542**	**MK773568**
*Kernia anthracina*	CGMCC 3.19002	Dung of marmot	Beijing, China	**MK773540**	**MK773546**	**MK773543**	**MK773569**
*Kernia anthracina*	CGMCC 3.19003	Dung of marmot	Beijing, China	**MK773541**	**MK773547**	**MK773544**	**MK773570**
*Kernia columnaris*	CBS 159.66T	Dung of hare	South Africa	**MN991957**	**MN982416**	**MN991962**	**MN982409**
*Kernia geniculotricha*	CBS 599.68T	On dung of *Oryctolagus cuniculus*	Germany	**MN991956**	**MN982414**	**MN991964**	**MN982408**
*Kernia hippocrepida*	CBS 774.70T	On dung of *Erethizon dorsatus*	Ontario, Canada	**MN991954**	**MN982413**	**–**	**MN982406**
*Kernia nitida*	CBS 282.52T	*Chrysolina sanguinolenta*	France	**MN991955**	**MN982415**	**MN991963**	**MN982407**
*Kernia pachypleura*	CBS 776.70T	On dung of *Loxodonta africana*	Uganda	**MN991958**	**MN982417**	**MN991965**	**MN982410**
*Microascus cirrosus*	CBS 217.31T	Leaf of *Prunus* sp.	Italy	KX923838	**–**	AF400860	**–**
*Microascus longirostris*	CBS 196.61T	Wasp's nest	USA: Maine	LM652421	LM652634	LN851043	LM652566
*Microascus senegalensis*	CBS 277.74T	Mangrove soil	Senegal	KX923929	**–**	AF400867	**–**
*Petriella musispora*	CBS 745.69	On rotten wood of *Populus grandidentata*	Ontario, Canada	MH859407	**–**	AF027663	**–**
*Petriella setifera*	CBS 390.75	skin lesion in Tursiops truncatus	Netherlands	AY882353	**–**	AF027664	**–**
*Petriellopsis africana*	CBS 311.72T	Brown sandy soil	Namibia	AJ888425	**–**	EF151331	**–**
*Pseudallescheria ellipsoidea*	CBS 418.73T	Soil	Tajikistan	EF151323	MH271617	AF027671	**–**
*Wardomyces anomalus*	CBS 299.61T	Air cell of egg	Canada: Ontario	LN850992	LN851149	LN851044	LN851095
*Wardomyces inflatus*	CBS 367.62T	Greenhouse soil	Belgium	LN850994	LN851153	AF400886	LN851099
*Wardomyces pulvinatus*	CBS 112.65T	Salt-marsh	England, UK	LN850997	LN851156	LN851051	LN851102

CBS: CBS Fungal Biodiversity Centre, Utrecht, The Netherlands.

Sequences newly generated in this study are indicated in bold.

‘T’ represents type strain.

### Phylogeny of two genera and genealogical concordance analysis

**Figure 3:**
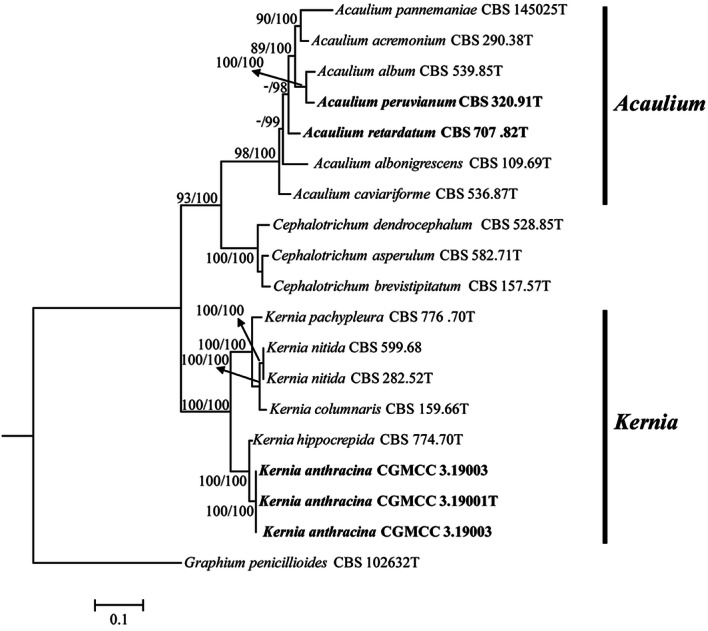
Maximum likelihood (ML) tree obtained from the combined *LSU*, *ITS*, *TEF* and *TUB* sequences of 19 representative taxa included most of species in the genera of *Kernia* and *Acaulium*. Numbers on the branches are ML bootstrap values (bs) above 75% and Bayesian posterior probabilities (pp) above 95%. A dash (–) indicates support value lower than 75% bs or 95% pp. Branch lengths are proportional to distance. Ex-type strains are indicated with T. The tree was rooted to *Graphium penicillioides* (CBS 102632).

The second dataset was composed of 19 taxa (including the outgroup) with the following four loci combined: *ITS* (1–434), *LSU* (435–1,163), *TEF* (1,164–2,024), *TUB* (2,025–2,401). The phylogenic tree was constructed and the branch support values (≥ 50%) and the Bayesian posterior probabilities from Bayesian analyses (≥ 95%) were indicated (Fig. 3). The phylogenetic tree grouped 19 strains into three clades comprising *Kernia* (bs = 100%/pp = 100%) and *Acaulium* (bs = 98%/pp = 100%) subclades with high bootstrap values. Coupled with morphological characteristics (Fig. 2), two new combination species *A. peruvianum* and *A. retardatum* are proposed. Besides, Three *Kernia* strains were clustered with the species of *K. hippocrepida* in independent group and have a well-supported value (bs = 100%/pp = 100%).

**Figure 4:**
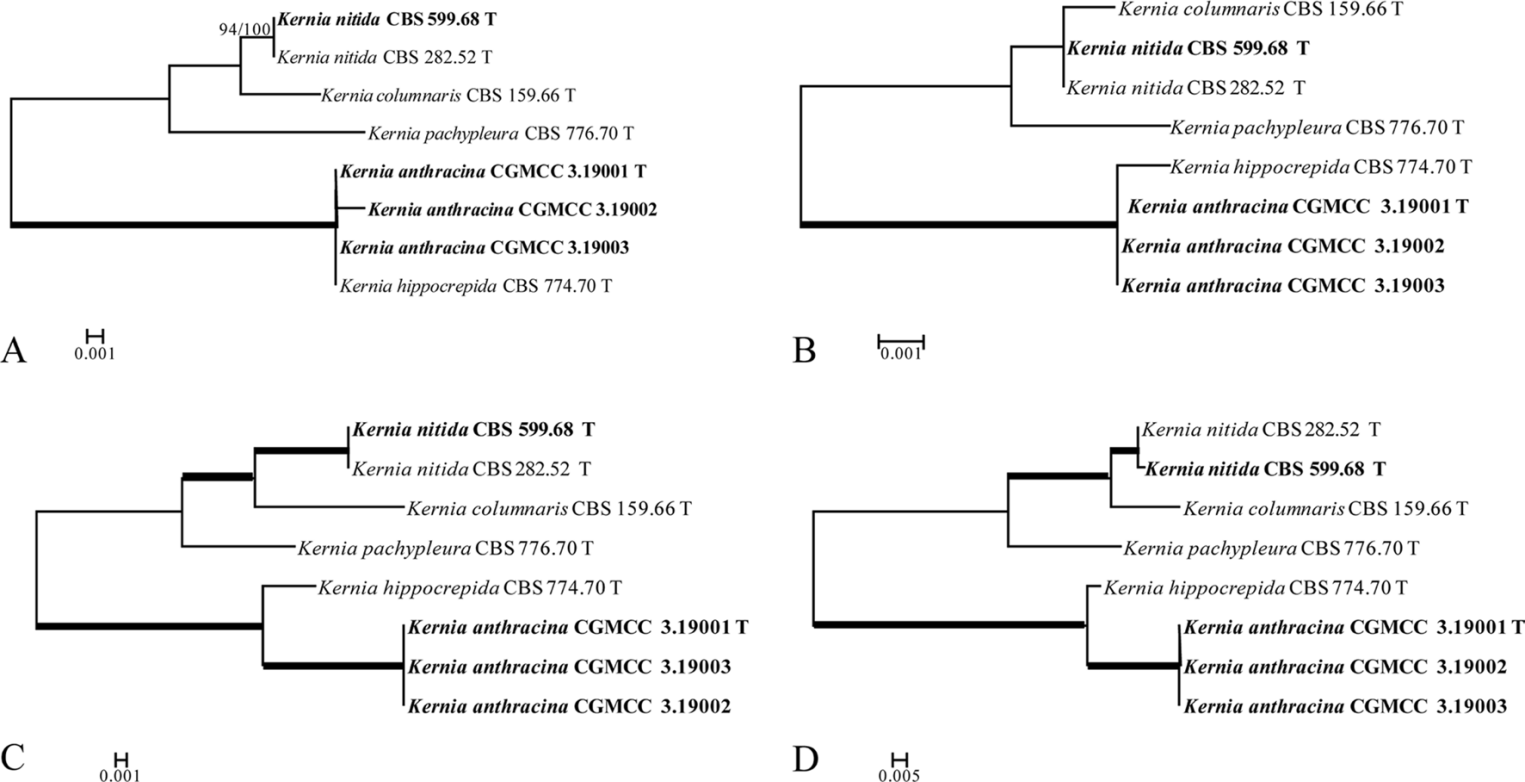
Delimitation of *Kernia anthracina* and its closely related species based on the separate analyses of four loci: *ITS* (**A**), *LSU* (**B**), *TEF* (**C**) and *TUB* (**D**). ML bootstrap values (bs) above 97% and Bayesian posterior probabilities (pp) above 99% are represented as bold lines. The *TUB* and *TEF* tree divides *K. anthracina* and *K. hippocrepida* isolates into different clades while the *ITS* loci place these isolates on a single branch. Genealogical concordance is seen in four trees, which supports *K. anthracina* and *K. hippocrepida* as distinct species.

BLAST searches of GenBank using the *ITS* sequences of three *Kernia* strains isolated from the dung of marmot revealed that three strains showed 94.7% similarity to *K. nitida* CBS 282.52T (KY852476). Multiple sequence alignment and sequence polymorphism analysis were further carried out in the *ITS* region of three *Kernia* strains. Compared with *K. hippocrepida* CBS 774.70T, had only two variable positions that is transition and indel in the *ITS* region. However, sequence polymorphism analysis in the *TUB* region indicated that strain CGMCC 3.19001T have 35 variable positions, including 12 transitions, 7 transversions, and 16 indels, indicating a lower similarity (93.2%) to *K. hippocrepida* CBS 774.70T (Supplementary Table 1). In genealogical concordance analysis, *K. hippocrepida* and *K. anthracina* strains were divided into different statistically supported subclades in *TUB* and *TEF* tree with a high bootstrap value (bs = 100%/pp = 100%) (Fig. 4). In addition, *K. geniculotricha* CBS 599.68T and *K. nitida* CBS 282.52T were clustered together and have a high similarity (99%) from four different gene tree.

### Taxonomy

**Figure 5:**
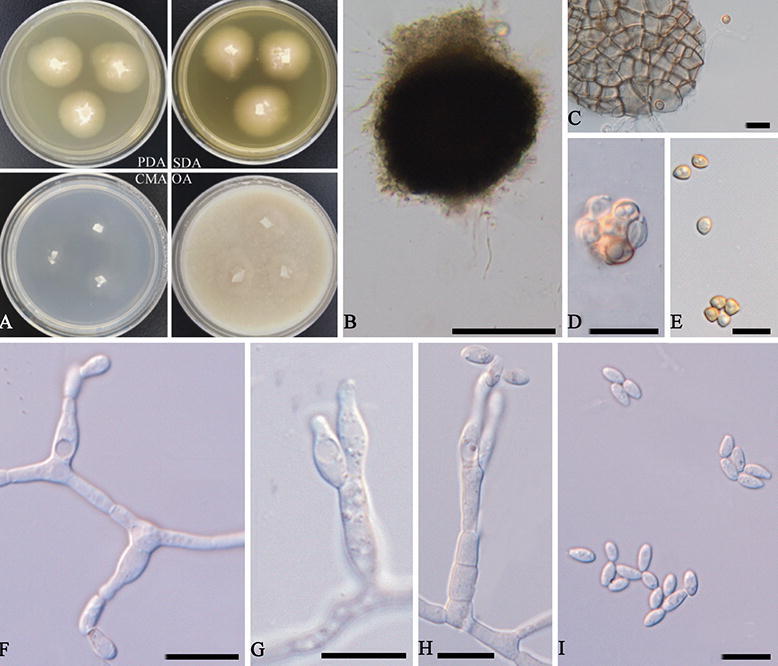
*Acaulium peruvianum* (ex-type CBS 320.91). (**A**) Colonies on different media after 10 days at 20 °C. (**B**) Ascoma. (**C**) Ascomatal peridium. (**D**,**E**) Ascospores. (**F**–**H**) Conidiophores and conidiogenous cells. (**I**) Conidia. Scale bars: (**B**) = 100 μm; (**C**–**I**) = 10 μm.

Based on the results of the above multilocus sequence analysis and a morphological analysis, the species of the genera *Acaulium* and *Kernia* have been reassessed accordingly. Their current circumscription is revised and several new taxa and combinations are proposed as follows:

***Acaulium peruvianum*** (Udagawa & Furuya) L. Su **comb. nov.** Fig. 5.

MycoBank: MB 834193.

*Basionym*: *Kernia peruviana* Udagawa & Furuya, *Mycotaxon* 33: 295. 1988.

*Hyphae* hyaline to subhyaline, smooth-walled, 1–4 μm (*x̅* = 2.7 μm) wide. *Conidiophores* often arising from the substratum or from the aerial mycelium, branched or unbranched, septate, smooth, cylindrical, 9–25 × 2–4 μm (*x̅* = 14.0 × 3.0 μm). *Conidiogenous cells* solitary or more commonly united into synnemata, percurrent in conidiophores or produced on hyphae in laterally, flask-shaped, subhyaline and smooth-walled, 6–11 × 2–3.5 μm (*x̅* = 8.9 × 2.7 μm). *Conidia* ellipsoidal to fusiform, with a truncate base and rounded or bluntly pointed apex, subhyaline, smooth and slightly thick-walled, 3.5–7 × 1–3 μm (*x̅* = 5.0 × 2.2 μm). Sexual morph observed. *Cleistothecia* superficial, non-ostiolate, dark brown to black, globose, 119–160 μm (*x̅* = 143.9 μm) diam., glabrous at maturity except for a few hyphal attachments. *Asci* 8-spored, globose to ovoid, evanescent. *Ascospores* irregularly arranged, pale yellowish brown to brown, broadly ovoid to fusiform, 3–5 × 2–4 μm (*x̅* = 4.0 × 2.8 μm).

Colonies on PDA reaching 17 mm diameter after 10 days at 20 °C, planar, finely felty with tufts of mycelium in center, white to cream. On SDA reaching 21 mm diameter, planar, finely felty with tufts of mycelium in center, white to cream. On CMA reaching 16 mm diameter, planar, subhyaline. On OA reaching 15 mm diameter, planar to low convex, white to creamcoloured centre, margin discrete.

Specimens examined. Peru, Tamshiyacu, near Iquitos, T. Akiyama, from soil, 1987, S. Udagawa (culture ex-type CBS 320.91 = NHL 2,985).

Notes. This species was originally placed in *Kernia* based on morphological features of the well developed sexual morph^9^. In our phylogenetic analysis, the ex-type culture of *Acaulium peruvianum* grouped with high statistical support with species of *A. album*. *A. peruvianum* is morphologically different with *A. album*, *A. peruvianum* produces sexual and asexual morphs in culture, besides, most of conidiogenous cells directly produced from hyphae. However, *A. album* has abundant monoverticillate, irregularly biverticillate and terverticillate, or reduced to single conidiogenous cells^10^. Conidiogenous cells of *A. album* are smaller (6–8.5 × 2.5–3 μm) than *A. peruvianum*.

**Figure 6:**
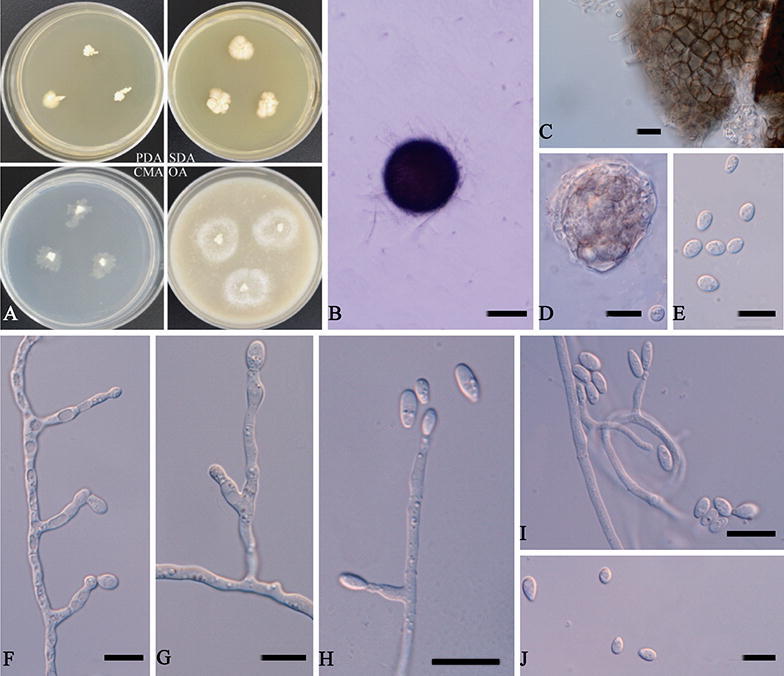
*Acaulium retardatum* (ex-type CBS 707.82). (**A**) Colonies on different media after 10 days at 20 °C. (**B**) Ascoma. (**C**) Ascomatal peridium. (**D**) Ascus. (**E**) Ascospores. (**F**–**I**) Conidiophores and conidiogenous cells. (**J**) Conidia. Scale bars: (**B**) = 100 μm; (**C**–**J**) = 10 μm.

***Acaulium retardatum*** (Udagawa & T. Muroi) L. Su **comb. nov.** Fig. 6.

MycoBank: MB 834194.

*Basionym*: *Kernia retardata* Udagawa & T. Muroi, *Trans. Mycol. Soc.* 22(1): 18. 1981.

*Hyphae* hyaline to subhyaline, thin- and smooth-walled, 1–4 μm (*x̅* = 2.6 μm) wide. *Conidiophores* branched or unbranched, septate, cylindrical. *Conidiogenous cells* flask-shaped to nearly cylindrical, subhyaline and smooth-walled, terminal or lateral in hyphae or hyphae coil, 8.5–23 × 2–5 μm (*x̅* = 13.3 × 3.0 μm). *Conidia* ellipsoidal to fusiform, with a truncate base, subhyaline, smooth and slightly thick-walled, 4–10.5 × 3–6 μm (*x̅* = 7.1 × 4.9 μm). Sexual morph observed. *Cleistothecia* superficial, non-ostiolate, dark brown to black, globose, 106.5–154 μm (*x̅* = 129.1 μm) diam., glabrous at maturity except for a few hyphal attachments. *Asci* 8-spored, globose to subglobose, evanescent. *Ascospores* irregularly arranged, grey to pale yellow, broadly ovoid to ellipsoidal, 4–8 × 3–5 μm (*x̅* = 5.6 × 3.7 μm).

Colonies on PDA reaching 5 mm diameter after 10 days at 20 °C, slow growing, raised centrally, with flat and irregular margin, white. On SDA reaching 9 mm diameter, moderately growing, raised centrally, aerial mycelium absent or sparse, white to cream. On CMA reaching 9 mm diameter, moderately growing, planar, white, margin discrete. On OA reaching 15 mm diameter, planar, white.

Specimens examined. Japan, Nishinasuno-machi, Nasu-gun, Tochigi, Udagawa, S, from rice-field soil, 1988, S. Udagawa (culture ex-type CBS 707.82 = NHL 2,879).

Notes. This species was originally placed in *Kernia* based on sexual morphological features^8^. The phylogenetic analysis shows that the ex-type culture of *Acaulium retardatum* grouped with statistical support with species of *A. albonigrescens* and *A. caviariforme*. *A. retardatum* is morphologically similar to *A. caviariforme*; both species produce sexual and asexual morphs in culture. However, *A. caviariforme* has fusiform, pale orange to copper-red ascospores, and brown, obovoid to ellipsoidal conidia^16^; ascospores of *A. retardatum* are smaller, broadly ovoid to ellipsoidal.

**Figure 7:**
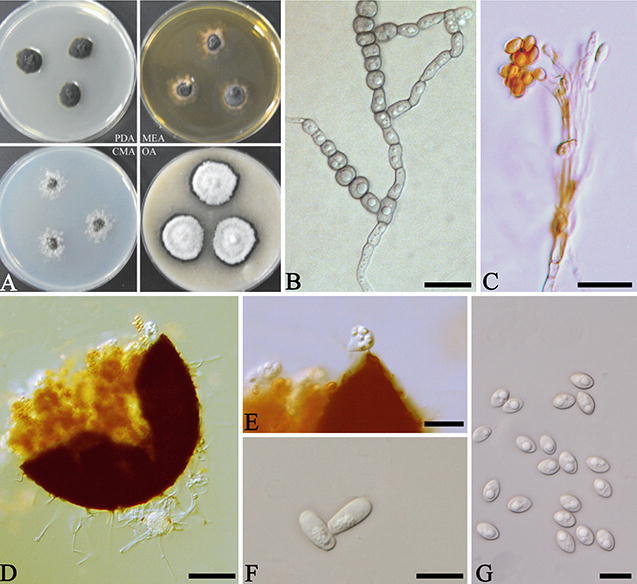
*Kernia anthracina* (CGMCC 3.19001). (**A**) Colonies on different media after 20 days at 30 °C. (**B**, **C**) Hyphae and conidiophores. (**D**) Cleistothecia. (**E**) Ascus. (**F**) Conidia. (**G**) Ascospores. Scale bars: (**B**,**F**, **G**) = 10 μm, (**C**–**E**) = 20 μm.

***Kernia anthracina*** L. Su, H. Zhu & C. Qin, **sp. nov.** Fig. 7.

MycoBank: MB 830661.

Etymology: Referring to the coal-black colony.

Holotype: HMAS 255463.

*Hyphae* septate, branched, catenate, hyaline to subhyaline, mostly 2–4.5 μm (*x̅* = 3.1 μm) wide. *Conidiophores*, with scopulariopsis-like branching pattern, produce acrospores. *Conidia* formed in slimy heads at the apex of the scopulariopsis-like branch, broadly clavate to ellipsoid with a slightly apiculate base, smooth to finely roughened, 6.5–14 × 1–5 μm (*x̅* = 10.8 × 3.2 μm). *Cleistothecia* abundant in CMA, gregarious, superficial, non-ostiolate, glabrous at maturity, black, globose to subglobose, 55–106 μm (*x̅* = 77.9 μm) diameter; peridium with a textura intricata. *Asci* ampulliform. *Ascospores* irregularly arranged, pale yellowish brown to straw coloured, ovoid to fusiform or ellipsoidal, 5–8 × 3–6 μm (*x̅* = 6.9 × 4.8 μm). Optimal growth temperatures are 25–30 °C, no growth at 40 °C.

Colonies on PDA reaching 6 mm diameter after 20 days at 30 °C, 4 mm on SDA, 5 mm on CMA and 13 mm on OA. Colonies on PDA, black in obverse, compact, reverse black, raised centrally, aerial mycelium absent or sparse.

Specimens examined. China, Beijing, Fangshan District, in north center for experimental animal resources, Institute of medical laboratory animal science, Chinese academy of medical sciences, 116°13′ E, 39°48′ N, 58 m above sea level, from fresh dung samples of healthy *Marmota monax*, 7 December 2017, collected and isolated by L. Su (HOLOTYPE: HMAS 255463, Institute of Microbiology, Chinese Academy of Sciences, Beijing, China; dried culture of ex-type CGMCC 3.19001T on PDA), living cultures, CGMCC 3.19001, CGMCC 3.19002, CGMCC 3.19003.

**Figure 8:**
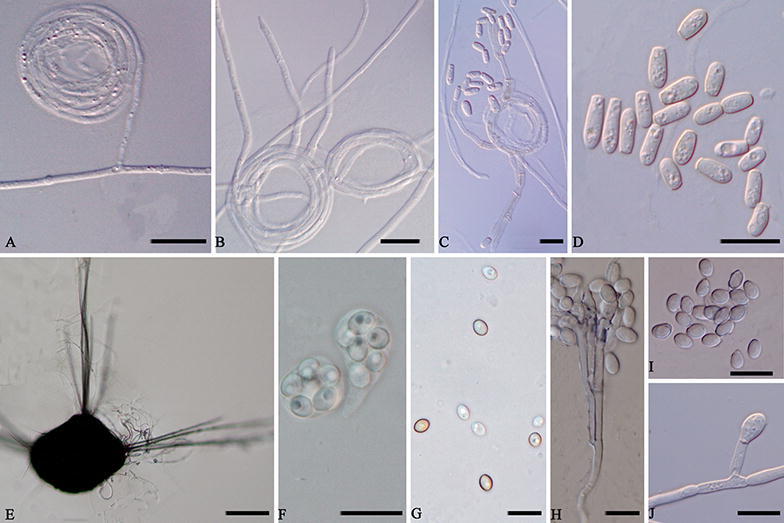
Morphology characters of the two species. *Kernia hippocrepida* (CBS 774.70). (**A**,**B**) Hyphae coil. (**C**) Conidiophores. (**D**) Conidia. *Kernia nitida* (CBS 599.68). (**E**) Cleistothecia. (**F**) Ascus. (**G**) Ascospores. (**H**) Conidiophores and conidiogenous cells. (**I**,**J**) Conidia. Scale bars: (**A**–**D**,**F**–**J**) = 10 μm; (**E**) = 100 μm.

Notes. Strains of *K. anthracina* have the typical features of *Kernia* such as compact growth, nonostiolate, ascomata, and ovoid to ellipsoidal ascospores^28^. *K. anthracina* not only has a scopulariopsis-like asexual morph, but is also supported by phylogenetic tree based on the combined four genes dataset (Fig. 3) and genealogical concordance analysis (Fig. 4). Although *K. anthracina* is closed to *K. hippocrepida* CBS 774.70T with *ITS* sequences, they apparently differs from morphological characters as *K. hippocrepida* produced reniform ascospores and conidiophores produced from coiled or irregularly twisted (Fig. 8A–D) while *K. anthracina* production of scopulariopsis-like conidiophores and ovoid to ellipsoidal ascospores.

*Kernia nitida* (Saccardo) Nieuwland. Amer. Midland Natur. 4: 379. 1916. Figure 8E–J.

*Basionym*: *Magnusia nitida* Saccardo. Michelia, 1: 123. 1878.

*Synonym*: *Kernia geniculotricha* Seth. Acta Bot. Neerl. 17: 481. 1968.

Description and illustrations: Seth (1968).

Specimens examined. Germany, near Hamburg, dung of rabbit, 1968, H.K. Seth (culture ex-type CBS 599.68 = ATCC 18529).

Notes. Although Malloch and Cain^28^ placed the species of *K. geniculotricha* as the synonym of *K. nitida* only based on numerous drawings and aquarels which are thus proposed, the isolate CBS 599.68 studied and proposed here conforms to the morphological characteristics of descriptions and phylogeny of *K. nitida*. The isolate of CBS 599.68 forms compact colonies on PDA, simple or branched, hyaline to light brown conidiophores, and bearing a cluster of annellophores directly at the apex or repeatedly and compactly branching to form a dense penicillus, 10–20.5 × 1–3.5 μm (*x̅* = 14.4 × 2.0 μm), produced in clusters of two to three at the tips of the conidiophores or metulae, rarely solitary, flaskshaped to nearly cylindrical, 7–15 × 1–4 μm (*x̅* = 9.7 × 2.5 μm); conidia ovoid to ellipsoidal, 3–6.5 × 2.5–4.5 μm (*x̅* = 5.1 × 3.2 μm). Besides, The isolate has a sexual morph characterised by abundant cleistothecia on CMA, gregarious, superficial, non-ostiolate, black, opaque, ovoid, 143–323.5 μm (*x̅* = 203.9 μm) diam., hairs emerging as two opposing or triangle symmetrical on the cleistothecium, dark brown to black. Asci 8-spored, globose to ampulliform, 8–18 μm (*x̅* = 11.2 μm) diam, evanescent. Ascospores irregularly arranged, pale brown, smooth, broadly ovoid to globose, 3.5–6 × 2.5–5.5 μm (*x̅* = 4.9 × 3.6 μm). In addition, *K. geniculotricha* is a well-circumscribed species described from on dung of *Oryctolagus cuniculus* in Germany. All these characters are similar to the species of *K. nitida*. Combined with phylogeny and genealogical concordance analysis, we identified the *K. geniculotricha* as the synonym of *K. nitida*.

### Identification keys

According to the morphological features, identification keys were constructed for the different genera including all the phylogenetic species recognised in this study (Supplementary information 1).

## Discussion

The family Microascaceae was established by Luttrell^26^, comprising saprobic and plant pathogenic species. Some species of Microascaceae are opportunistic pathogens and show intrinsic resistance to antifungal agents^11, 20, 27^. Recent molecular studies have demonstrated that the Microascaceae contains several closely related genera that are difficult to separate morphologically^11^ including *Microascus*, *Scedosporium* and *Scopulariopsis*. Recently, three of the most debated genera of the family, *Microascus*, *Scopulariopsis* and *Pithoascus* were revised by morphology and multigene phylogeny^11, 16^. As a result, several taxa were excluded from these genera and appeared as a new lineage within the Microascaceae as *Acaulium*^11^.

In this study we have reviewed the taxonomic circumscription of species in the genera of *Kernia* and *Acaulium*, traditionally referred to as sexual and asexual morphs, respectively, and two genera using a polyphasic approach based on the genealogical concordance analysis, phylogeny and morphological data. These results show that *Kernia* and *Acaulium* constitute two phylogenetically distant lineages, combining the results of phenotypic data, delineate the accepted species of the two new combination species, proposing a new species, which are clarifying the identity of species as *Kernia nitida* and *K. geniculotricha*, reclassifying the white synnematous species as *Acaulium peruvianum* and *A. retardatum*, and describing a new species *K. anthracina* isolated from the dung of marmot. The species of *Kernia* are mainly isolated from dung of various animals, while the species of *Acaulium* have a worldwide distribution and are mainly isolated from dung, litter, soil, skin of a horse and decaying meat^4–10, 16^.

Sandoval-Denis et al.^11^ was firstly attempts to clarify phylogenetically the relationships among the different genera of the Microascaceae by the use of partial *LSU* and *ITS* sequences. Subsequently, Microascaceae was revised by Sandoval-Denis et al.^16^ based on morphological, physiological and molecular phylogenetic analyses using DNA sequence data of four loci (*ITS*, *LSU*, *TEF* and *TUB*). These studies demonstrated that several genera of Microascaceae raised questions concerning correct positions of several members of the family and their generic circumscriptions, suggesting a possible subdivision of *Microascus* and *Scopulariopsis* into several smaller genera as *Kernia* and *Acaulium.* Our results based on the phylogenetic reconstructions of two loci (*LSU*, *ITS*) indicated that *Kernia* and *Acaulium* fall into two groups (Fig. 1). Besides, it also shows that *Kernia* and *Acaulium* species can be well separate by phylogentic analysis of four loci (*LSU*, *ITS*, *TEF* and *TUB*). As known that *Acaulium* is characterised by the formation of pale colonies with dense hyphal fascicles and the presence of abundant oil drops in the mycelium, conidia and ascospores, showing a guttulate appearance^16^. The new combination species *A. peruvianum* and *A. retardatum* clustered in *Acaulium* group, in which the species produce white and pale grey colonies, and have a wide isolation source. A new species was clustered in *Kernia* group, which form compact dark brown or black colonies, and mainly isolated from dung (Figs. 2, 3). In addition, species delineation was also assessed in the genus of *Kernia* as the closed species of *K. anthracina* and *K. hippocrepida* under the genealogical concordance analysis using DNA sequence data of four loci (Fig. 4).

The absence of clear diagnostic morphological characters can be used to identify species which belonging to the *Kernia* and *Acaulium* species^2, 11, 16^. The species of ‘*K. geniculotricha*’ and *K. nitida* have been identified two different species according to morphologically characters, but molecular data can easily identified them as the same species, using any of the four genes studied here. Some species as *A. peruvianum* and *A. retardatum* isolates were initially identified as *K. peruviana*, *K. retardate* at CBS based on their morphology. Combined the molecular data, the group of species that would previously have been included in *Kernia* and *Acaulium* are easily recognized. Our phylogeny demonstrates that, although *Kernia* and *Acaulium* share similar morphological and ecological traits, they are in fact genetically distant. The phylogenetic data is supported by relevant morphological differences, such as the color of colonies, the shape of ascospores or conspicuously hairy ascomata^2, 8–10^.

The new species, *K. anthracina* and *K. hippocrepida*, are very similar in *ITS* but easily distinguished by *TUB* and *TEF* sequences. All *Kernia* species can be well separated with *TUB* and *TEF* partial gene sequences. Based on *ITS* alone, *K. anthracina* and *K. hippocrepida* cannot be distinguished (Fig. 4), but morphology and *TUB* and *TEF* sequences clearly differentiate them. The lack of the isotype herbarium specimens examined here prevented us from conclusively characterizing five of the other described species *K. bifurcotricha*, *K. setadispersa*, *K. cauquensis*, *K. irregularis*, *K. ovata*, leaving them as nomena dubia. From our study, we found that it is easy to identify the species of *Kernia* and *Acaulium* by polyphasic approach.

The delimitation of the two genera in this study contributes to an integrated phylogeny of the family Microascaceae. The two monophyletic genera currently accepted are statistically supported in the four-locus phylogeny (Fig. 3). There are seven species included in *Acaulium* by our revision, while ten species in *Kernia*. It is regret that some species of *Kernia* absent holotype material and unavailable for these species. Therefore, further studies are needed to establish a comprehensive modern classification of the *Kernia* and to give better insight into the evolutionary relationships among the species in the genus.

## Materials and methods

Eight *Kernia* and *Acaulium* ex-type strains were obtained from the CBS culture collection (CBS) housed at the Westerdijk Fungal Biodiversity Institute (WI), Utrecht, the Netherlands. More isolates potentially related to the obtained *Acaulium* strains were selected based on a preliminary phylogenetic analysis of *LSU* + *ITS* sequences from GenBank, as well as several cultures of the *Kernia*, which isolated from the feces of *Marmota monax*^25^, maintained in China General Microbiological Culture Collection Center (CGMCC) in China. All the strains used in this study are listed in Table 1. The strains were incubated on different media such as Potato dextrose agar (PDA), Malt extract agar (MEA), Sabouraud Dextrose Agar (SDA), Corn meal agar (CMA), and Oatmeal agar (OA) (Becton, Dickinson & Co.) at 20 °C. Colony morphology and microscopic characteristics were examined, measured and photographed after incubation for 10 days with the methods of Su et al.^25^. Means and standard deviations (SD) were calculated from at least 50 measurements. The ex-type living cultures were deposited in the China General Microbiological Culture Collection Center (CGMCC). The dried culture and microscope slide were deposited in Herbarium Mycologicum, Academia Sinica, Beijing, China (HMAS). Nomenclatural novelties and descriptions were registered in MycoBank (https://www.MycoBank.org).

### DNA extraction, PCR amplification and sequencing

Total genomic DNA was extracted from mycelia grown on PDA or OA plates using the protocol of Guo et al.^28^. Primers ITS1 and ITS4 were used to amplify the *ITS* region of the nuclear rRNA gene^29^, primers LROR/LR5 primers were used for the partial 28S rDNA (*LSU*)^30^, primers 983F and 2218R^31^ for the elongation factor 1-α gene (*TEF*), and primers Bt2a and Bt2b^32^ for the partial β-tubulin gene (*TUB*). PCR was performed in a 25 μL reaction volume containing 1.0 μL DNA template, 1.0 μL of each forward and reverse primers, 12.5 μL 2 × MasterMix (Tiangen Biotech Co. Ltd., Beijing, China) and 10.5 μL ddH_2_O with the following cycling parameters: 94 °C for 40 s; 35 cycles at 94 °C for 40 s, annealing temperature specific for the gene amplified (52 °C for *LSU*, 55 °C for *TEF* and *ITS*, 58 °C for *TUB*) for 60 s and 72 °C for 120 s; and a final extension at 72 °C for 10 min. The PCR products were sequenced by Beijing Sunbiotech Co. Ltd. (Beijing, China). Sequences were compared with accessions in the GenBank database via a BLASTn search to determine the most likely taxonomic designation.

### Phylogenetic analysis

Sequence data of the four loci were aligned with Clustal X^33^. Reference sequences were retrieved from GenBank and the accession numbers indicated in Table 1. Manual editing of sequences was performed in MEGA6^34^. The concatenated sequences (*LSU* + *ITS*) or (*LSU* + *TUB* + *TEF* + *ITS*) were assembled using SeaView^35^ and alignments were deposited in TreeBASE (www. treebase.org, submission no.: S25764). The combined dataset of two or four loci was analyzed phylogenetically using Bayesian MCMC^36^ and Maximum Likelihood^37^, respectively. For the Bayesian analyses, the models of evolution were estimated by using MrModeltest 2.3^38^. Posterior probabilities (PP)^39,40^ were determined by Markov Chain Monte Carlo sampling (MCMC), Six simultaneous Markov chains were run for 2,000,000 generations and trees were sampled every 100th generation (resulting in 20,000 total trees). The first 4,000 trees represented the burn-in phase of the analyses and were discarded and the remaining 16,000 trees were used for calculating PP in the majority rule consensus tree. For the ML analysis in RAxML^37^, the GTRGAMMA model was used for all partitions, in accordance with recommendations in the RAxML manual against the use of invariant sites. Analyses were performed using the CIPRES web portal^41^. Trees were visualised in TreeView 1.6.6^42^.

## Supplementary information

Supplementary information 1 (DOCX 14 kb)

Supplementary information 2 (DOCX 13 kb)
