# Author Correction: Phylogeny and taxonomic revision of *Kernia* and *Acaulium*

**DOI:** 10.1038/s41598-020-71842-w

**Published:** 2020-09-10

**Authors:** Lei Su, Hua Zhu, Yongchun Niu, Yaxi Guo, Xiaopeng Du, Jianguo Guo, Ling Zhang, Chuan Qin

**Affiliations:** 1 NHC Key Laboratory of Human Disease Comparative Medicine, Institute of Medical Laboratory Animal Science, Chinese Academy of Medical Sciences (CAMS), Beijing, China; 2 Beijing Engineering Research Center for Experimental Animal Models of Human Critical Diseases, Chinese Academy of Medical Sciences (CAMS), Beijing, China; 3 Key Laboratory of Microbial Resources, Ministry of Agriculture/Institute of Agricultural Resources and Regional Planning, Chinese Academy of Agricultural Sciences, Beijing, 100081 China

Correction to: *Scientific Reports* 10.1038/s41598-020-67347-1, published online 25 June 2020.

This Article contains errors in Table 1.

In the LSU column, the GenBank accession number for *Acaulium peruvianum*,

“MN991966”

should read:

“MN991967”

In the same column, the GenBank accession number for *Kernia hippocrepida* was omitted, and should read “MN991966”

