# Supplementary material for: Phylogeny and taxonomic revision of Kernia and Acaulium
Source: Sci Rep. 2020 Jun 25;10:10302. doi: 10.1038/s41598-020-67347-1 (PMC7316793; doi:10.1038/s41598-020-67347-1)
Supplement: Supplementary file 1 — Supplementary information 1 (DOCX 14 kb) [file 41598_2020_67347_MOESM1_ESM.docx]

**Full Paper**

**Phylogeny and taxonomic revision of *Kernia* and *Acaulium***

Lei Su^1,2^ ∙ Hua Zhu^1,2^ ∙ Yongchun Niu^3^ ∙ Yaxi Guo^1,2^ ∙ Xiaopeng Du^1,2^ ∙ Jianguo Guo^1,2^ ∙ Ling Zhang^1,2^ & Chuan Qin^1,2*^

*^1^ NHC Key Laboratory of Human Disease Comparative Medicine,* *Institute of Medical Laboratory Animal Science, Chinese Academy of Medical Sciences (CAMS), Beijing, China*

*^2^ Beijing Engineering Research Center for Experimental Animal Models of Human Critical Diseases, Chinese Academy of Medical Sciences (CAMS), Beijing, China*

*^3^**Key Laboratory of Microbial Resources, Ministry of Agriculture/Institute of Agricultural Resources and Regional Planning, Chinese Academy of Agricultural Sciences, Beijing 100081, China*

*Correspondence author: Chuan Qin

*E-mail: qinchuan@pumc.edu.cn*

**Key to *Acaulium* species**

1a. Colonies raised, muddy, aerial mycelium absent, white to yellowish on PDA..2

b Colonies expanding, white to grey-white on PDA…………………..………..4

2a. Sexual morph absent; conidia obovate up to 12 μm long ...................................*A. acremonium*

b Sexual morph present …………………………………………………..….....3

3a. Ascospore fusiform, pale orange to copper-red ascospores…………….……...*A. caviariforme*

b Ascospore broadly ovoid to ellipsoidal, grey to yellowish……………………*A. retardatum*

4a. Conidiophores tall, ramose, usually with secondary branches.............................5

b Conidiophores usually without secondary branches…………...……………....6

5a. Conidia obovoid, ellipsoidal to irregularly fusiform…….....................................*A. album*

b Conidia bullet-shaped or broadly clavate…..........................................................*A. pannemaniae*

6a. Ascospore lunate with rounded ends; conidia cylindrical to clavate……........... *A. albonigrescens*

b Ascospore broadly ovoid to fusiform; conidia ellipsoidal to fusiform………... *A. peruvianum*

**Key to *Kernia* species**

1a. Colonies white to yellowish coloured on PDA; ascospores reniform……….…..*K. hippocrepida*

b Colonies grow slowly, tan or brown to black coloured on PDA………………...2

2a. Cleistothecium has long hairs in two opposing tufts or symmetrical triangle........*K. nitida*

b Cleistothecium without such appendages ..............................................................3

3a. Asci ampulliform......................................................................................................*K. anthracina*

b Asci ovoid to globose..............................................................................................4

4a. Conidiophores mononematous or rarely synnematous with poorly developed…...*K. columnaris* bConidiophores compactly branching to form Scopulariopsis conidial state..........*..K. pachypleura*
