# Supplementary material for: Phylogeny and taxonomic revision of Kernia and Acaulium
Source: Sci Rep. 2020 Jun 25;10:10302. doi: 10.1038/s41598-020-67347-1 (PMC7316793; doi:10.1038/s41598-020-67347-1)
Supplement: Supplementary file 2 — Supplementary information 2 (DOCX 13 kb) [file 41598_2020_67347_MOESM2_ESM.docx]

**Full Paper**

**Phylogeny and taxonomic revision of *Kernia* and *Acaulium***

Lei Su^1,2^ ∙ Hua Zhu^1,2^ ∙ Yongchun Niu^3^ ∙ Yaxi Guo^1,2^ ∙ Xiaopeng Du^1,2^ ∙ Jianguo Guo^1,2^ ∙ Ling Zhang^1,2^ & Chuan Qin^1,2*^

*^1^ NHC Key Laboratory of Human Disease Comparative Medicine,* *Institute of Medical Laboratory Animal Science, Chinese Academy of Medical Sciences (CAMS), Beijing, China*

*^2^ Beijing Engineering Research Center for Experimental Animal Models of Human Critical Diseases, Chinese Academy of Medical Sciences (CAMS), Beijing, China*

*^3^**Key Laboratory of Microbial Resources, Ministry of Agriculture/Institute of Agricultural Resources and Regional Planning, Chinese Academy of Agricultural Sciences, Beijing 100081, China*

*Correspondence author: Chuan Qin

*E-mail: qinchuan@pumc.edu.cn*

| Supplementary Table 1. The difference between *Kernia hippocrepida* CBS 774.70T and *K. anthracina* CGMCC 3.19001T from the sequence alignment. | | | | |
| --- | --- | --- | --- | --- |
| Base mutation | ITS | LSU | TUB | TEF |
| Transitions | 1 | 2 | 12 | 7 |
| Transversions | 0 | 0 | 7 | 7 |
| Indels | 1 | 0 | 16 | 2 |
